# Suppressive effects of processed aconite root on dexamethasone-induced muscle ring finger protein-1 expression and its active ingredients

**DOI:** 10.1007/s11418-022-01606-5

**Published:** 2022-02-18

**Authors:** Taishi Kondo, Tomoaki Ishida, Ke Ye, Marin Muraguchi, Yohei Tanimura, Masato Yoshida, Kan’ichiro Ishiuchi, Tomoki Abe, Takeshi Nikawa, Keisuke Hagihara, Hidetoshi Hayashi, Toshiaki Makino

**Affiliations:** 1 Department of Pharmacognosy, Graduate School of Pharmaceutical Sciences, Nagoya City University, 3-1 Tanabe-Dori, Mizuho-ku, Nagoya, Aichi 467-8603 Japan; 2 Healthy Food Science Research Group, Cellular and Molecular Biotechnology Research Institute, National Institute of Advanced Industrial Science and Technology (AIST), 1-1-1 Higashi, Tsukuba, Ibaraki 305-8566 Japan; 3 Department of Nutritional Physiology, Institute of Medical Nutrition, Tokushima University Graduate School, 3-18 Kuramoto-cho, Tokushima, 770-8503 Japan; 4 Department of Advanced Hybrid Medicine, Graduate School of Medicine, Osaka University, 2-2 Yamadaoka, Suita, 565-0871 Japan; 5 Department of Cell Signaling, Graduate School of Pharmaceutical Sciences, Nagoya City University, 3-1 Tanabe-Dori, Mizuho-ku, Nagoya, Aichi 467-8603 Japan

**Keywords:** Processed aconite root, Muscle ring finger protein-1, Muscular atrophy, Higenamine, Salsolinol

## Abstract

Processed aconite root (PA), the tuberous root of *Aconitum carmichaelii* prepared by autoclaving, is a crude drug used in Japanese traditional Kampo medicine and traditional Chinese medicine for the symptoms of *kidney* deficiency, that is related to the muscle atrophy in modern medicine. The objective of the present study is to evaluate the effectiveness of PA on muscle atrophy and to find its active ingredients using dexamethasone-induced muscle ring finger protein-1 (MuRF1) mRNA expression in murine myoblast C2C12 cells. Dexamethasone-induced MuRF1 expression was significantly suppressed by methanol-soluble part of boiling water extract of PA in a concentration-dependent manner with its IC_50_ value of 1.5 mg/ml. By the activity-guided fractionations of PA extract using the partition between organic solvents and its aqueous solution, the activity of PA did not transfer into the fraction containing aconitine-type diterpenoid alkaloids but into BuOH layer. Then, we found higenamine and salsolinol as the active ingredients in PA. Higenamine and salsolinol significantly suppressed dexamethasone-induced MuRF1 expression, and their IC_50_ values were 0.49 and 50 µM, respectively. The contents of higenamine and salsolinol in the decoctions of commercially available fourteen PA products are 0.12 and 14 µg/ml as the average values, and varied with the coefficient of variation (CV) values of 97 and 63%, respectively. Higenamine also significantly suppressed dexamethasone-induced mRNA expressions of muscle atrophy F-box protein (MAFbx)/atrogin1, casitas B-lineage lymphoma-b (Cbl-b), troponin, branched-chain amino acid aminotransferase 2 (BCAT2), and Bcl-2 binding and pro-apoptotic protein3 (Bnip3). Although the quality control of PA is regulated by the contents of diterpene alkaloids, salsolinol and higenamine can be used as the marker compounds to certificate the pharmacological activities of PA.

**Graphical abstract:**

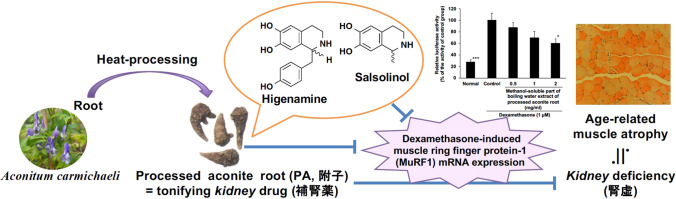

## Introduction

Sarcopenia and frailty, a syndrome of the loss of skeletal muscle mass and strength that occurs with aging, become a common medical and social topics in aging of the population [1]. In Japanese traditional Kampo medicine and traditional Chinese medicine, the physical problems related to aging are considered as the deficiencies of *kidney*, which is imaginary organ to stock the so-called life energy, and it is considered that adults have been able to live by using this energy of *kidney* [2, 3]. Goshajinkigan is one of the Kampo formulas to supply the *kidney* energy to treat *kidney* deficiencies, and used to treat low back pain [4], diabetic complications [5], and chemotherapy-induced peripheral neuropathy [6]. In our previous animal or in vitro studies, the extract of goshajinkigan has the ability to reduce sarcopenia symptoms in senescence-accelerated mouse P8 [7], to ameliorate allodynia in chronic constriction injury model mice [8] and in streptozotocin-induced diabetic model mice [9], and to suppresses voltage-gated sodium channel Nav1.4 current in murine myoblast C2C12 cells [10] and Nav1.7 current in HEK293 cells expressing Nav1.7 [9].

It is revealed that a muscle-specific ubiquitin ligase is one of the causative genes of skeletal muscle atrophy, and that the enhancement of proteolysis mainly due to the increased activity of the ubiquitin/proteasome system is greatly involved [11]. Muscle ring finger protein-1 (MuRF1) and muscle atrophy F-box protein (MAFbx)/atrogin-1 are the specific ubiquitin ligases in skeletal and cardiac muscle, and their expressions are increased in muscle atrophy caused by sciatic nerve transection [12]. These are considered to be important factors responsible for muscular atrophy, because the excessive MAFbx/atrogin-1 expression produced atrophy in myotubes, whereas mice with the deficiencies of either MAFbx/atrogin-1 or MuRF1 gene were resistant to muscular atrophy [13].

Among crude drugs composed in goshajinkigan, processed aconite root (PA), the tuberous root of *Aconitum carmichaelii* prepared by autoclaving, has the effectiveness of to supply *kidney* energy [14]. PA is considered to be one of the active components of goshajinkigan for the prevention from chemotherapy-induced peripheral neuropathy and mechanical hyperalgesia in diabetic mice, and its active ingredient containing in PA is neoline [9, 15, 16]. However, the preventive effects on muscular atrophy have not been evaluated.

In this study, we evaluated the effectiveness of PA extract on dexamethasone-induced MuRF1 expression in C2C12 cells in vitro and found the active ingredients contained in processed aconite root.

## Materials and methods

### Materials

Processed aconite root (lot: F2F0243) was purchased from Uchida Wakanyaku (Tokyo, Japan). Processed aconite root (10 g) was boiled in 200 ml of water for 30 min. After filtrating and freeze-drying, methanol was added into the lyophilized powder, and centrifuged (3 × 10^3^ rpm, 15 min), and the parts dissolved in methanol were taken to evaluate the activity. All the samples were dried up, and dissolved in DMSO. Extraction rates from the weight of each crude drug to methanol-soluble part were 13%.

### Isolation of salsolinol from PA extract

Processed aconite root (lot: F2F0243, Uchida; 1.0 kg) was boiled in 8 l of deionized water for 30 min. After filtering by gaze, the residue was further boiled, and this operation was repeated twice. After freeze drying the whole boiling water extract (373 g yielded), the powder was extracted with methanol for three times. After removal of the solvent under the reduced pressure, the resulting extract (124 g) was dissolved in 800 mL of acidified water at pH 3 with HCl and extracted with EtOAc (800 ml × 3). After adjusting the pH of water layer to 10 by adding NH_3_ solution, the water layer was extracted with EtOAc (800 ml × 3). After adjusting the pH of water layer was adjusted to 7 by adding HCl, the remained water layer was extracted with water saturated *n*-BuOH (800 ml × 3). The resulting extracts were concentrated under the reduced pressure to give acidic EtOAc (acidic layer, 4.0 g), alkaline EtOAc (alkaline layer, 4.2 g), *n*-BuOH (BuOH layer, 10 g), and water (water layer, 96 g) fractions. The BuOH layer (9.9 g) was subjected to silica gel (BW-200, Fuji Silysia, Fuji, Japan; 200 g) column chromatography and eluted with mobile phase using each 600 ml of CHCl_3_/MeOH mixture 10:1, 5:1, 3:1, 2:1, 1:1, 1:10, and 0:1, stepwise. At last silica gel column was washed by CHCl_3_/MeOH/H_2_O 6:4:1. The eluate was monitored by TLC (TLC Silica gel 60 F254; Merck, Kenilworth, NJ, USA) and combined to fraction (Fr.) 1 (0.39 g), Fr. 2 (1.1 g), Fr. 3 (1.1 g), Fr. 4 (1.1 g), Fr. 5 2.1 g), Fr. 6 (2.4 g), Fr. 7 (0.62 g), Fr. 8 (0.10 g), and Fr.9 (0.27 g). Fraction 3 (14 mg) was separated using preparative HPLC (column, Cosmosil 5C_18_-AR-II (10 i.d. × 250 mm), Nacalai Tesque, Kyoto, Japan; mobile phase, 5% acetonitrile (0–5 min), 5–100% acetonitrile (5–35 min), 4 ml/min), and from the peak eluted at 13.3 min, salsolinol (2.4 mg) was obtained and identified using NMR and MS spectrometry [17].

### Cell culture

C2C12 myoblasts were purchased from Bioresources Center (Tsukuba, Japan) and maintained and proliferated at 37 °C with 5% CO_2_ in Dulbecco's modified eagle medium (DMEM, Sigma-Aldrich, St. Louis, MO, USA), supplemented with 10% fetal bovine serum (FBS, Sigma), 100 U/ml penicillin, and 0.1 mg/ml streptomycin (Nacalai Tesque, Kyoto, Japan). At a confluence of 80%, C2C12 myoblasts were transferred to next generation by 0.25% trypsin (Sigma)/0.02%EDTA⋅2Na.

### Plasmid transfection and differentiation in C2C12 cells

C2C12 cells (4.0 × 10^6^ cells) were planted into 10 cm dish and maintained for one night. pGL3-MuRF1 was constructed in our previous study [18], and pCMVβ-gal was from Prof. Jeffrey L. Wrana [19]. After the medium was exchanged into FBS-free one, these genes were transfected into the cells using Hily Max^®^ (Dojindo, Tokyo, Japan) and Opti-MEM^®^ (Thermo Fisher Scientific, Waltham, MA, USA) according to the manufacturer’s instruction, and the cells were maintained for 8 h. Then, the cells were harvested with 0.25% trypsin/0.02%EDTA⋅2Na, transplanted into 24-well plate (4.0 × 10^4^ cells/well), and maintained for overnight. Then, the medium was exchanged into DMEM containing 2% horse serum (Sigma), 100 U/ml penicillin, and 0.1 mg/ml streptomycin (differentiation medium to induce myotube formation), and the cells were incubated for 72 h.

### Luciferase assay for MuRF1 induction

The MuRF1-transfected differentiated cells were incubated with the medium containing dexamethasone (Sigma) (1 µM) with/without the extract of PA, ( ±)-salsolinol hydrochloride (Cayman Chemical, Ann Arbor, MI, USA), and higenamine (Chengdu Must Bio-Technology, Chengdu, China) for 24 h. There are no standard drugs to use sarcopenia in clinics; the positive control cannot be used in this experiment. After washing cell surfaces by phosphate-buffered saline, the cells were lysed with 80 µl/well of lysis buffer [20 mM dithiothreitol, 2 mM EDTA, 10% glycerol, 1% Triton X-100 in phosphate buffer (0.2 M. pH 7.5)] by shaking the plates for 30 min at room temperature. the lysates (25 µl) were transferred into 96-well white plate and reacted with 50 µl of the luciferase assay regent (20 mM tricine (Sigma), 0.05% magnesium carbonate basic (Nacalai), 2.5 mM MgSO_4_, 10 µM EDTA, 0.53 mM ATP Mg (Sigma), 33 mM dithiothreitol, 270 µM coenzyme A trilithium salt from yeast (Fujifilm Wako Pure Chemicals, Osaka, Japan), and 0.45 mM luciferin K (Fujifilm) in 0.1 M phosphate buffer (pH 8.0). And then, the luminescence signals of all wells were measured using a microplate reader (Wallac 1420 Workstation, PerkinElmer, Waltham, MASS, USA). Otherwise, the lysate (10 µl) was transferred to 96-well plate and reacted with 80 µl of the ONPG regent (1.1 mM MgCl_2_, 3.2 µM *O*-nitrophenyl-β-d-galactopyranoside (Fujifilm), 0.71 mM 2-mercaptoethanol in 0.1 M phosphate buffer, pH 7.5) for 30 min at room temperature. The absorbance at 405 nm of all wells were measured by a plate reader. By calculating the amount of the level of luminescence signals divide by the level of absorbance at 405 nm, the luciferase activity was obtained. Data are expressed as the percent of the activity of control group in graphs. The percent of the inhibition in each data were calculated by the following formula: (the luciferase activity − the average value of normal group)/[(the average value of control group) − (the average value of normal group)] × 100, and the half-maximal inhibitory concentration (IC_50_) was calculated from the least square regression line made from 3 points that crossed at 50% of the percent of the inhibition value and the logarithmic concentration values.

### MTT assay

Differentiated C2C12 myoblasts (1 × 10^4^/well) were plated in a 96-well plate, treated with the medium containing dexamethasone with/without the sample, and incubated for 24 h. After the surface of the cells was rinsed with PBS, the medium containing MTT (Nacalai, 50 µg in 100 µl) was added to the cells, and further incubated for 4 h. After the cells were rinsed with PBS, DMSO (100 µl) was added to each well to dissolve the resulting formazan, and the absorbance at 570 nm was measured.

### Quantitative real-time polymerase chain reaction

Differentiated C2C12 cells were planted into 24-well plate (4.0 × 10^6^ cells/well) and incubated with the medium containing higenamine with or without dexamethasone (1 µM) for 24 h. After washing cell surfaces by phosphate-buffered saline, total RNA was extracted using RNA iso plus (Takara Bio, Shiga, Japan), and reverse transcribed to first-strand cDNA using PrimesScriptTM™ Master Mix (Takara Bio) according to the manufacture’s instruction. Quantitative real time PCR was performed in StepOne Real-time PCR system using twofold diluted Power SYBR Green PCR Master Mix (Applied Biosystems, Foster City, CA, USA). The primer sequences used are shown in Table 1. Relative quantification of target gene was calculated using the − 2^ΔΔ*Ct*^ method. Data are expressed as fold changes of the target gene/glyceraldehyde-3-phosphate dehydrogenase (GAPDH) compared with those of the control.

**Table 1:** List of oligonucleotide primer pairs used in RT-qPCR

Gene	Forward	Reverse	Product size (bp)
MuRF1	ACGAGAAGAAGAGCGAGCTGCT	TCCCTGTACTGGAGGATCAGA	91
MAFbx/atrogin-1	ACCCATGCAGGACTCCCAGACTTA	AGCCACACCCCTCTTGCTTTTG	87
Cbl-b	AAGTGGCCAAGTTCCATTGC	TGCGAACCATCGGAAGATGA	111
Troponin	AGGCTATGTCTGGCATGGAA	GAGTGTCATACAGCAAGCCA	92
MyHC	ACCTTCAGCTCTGAGTTTGC	ACGCTTCTGGAGCTTAAGGA	111
IGF1	TCCTTCTCAAGCCTGAGGTT	GGTTAGCAATGCCCAGTTGA	98
Bnip3	CTGCACTTCAGCAATGGCAA	ATGCTGGGCATCCAACAGTA	93
BCAT2	TGGCTCAACATGGACAGGAT	TCAATGAGCTGGCGGATACA	98
GAPDH	CAAGATTGTCAGCAATGCATCC	CCTTCCACAATGCCAAAGTTG	87

*MuRF1* muscle ring finger protein-1, *MAFbx* muscle atrophy F-box protein, *Cbl-b* casitas B-lineage lymphoma-b branched-chain, *MyHC* myosin heavy chain, *IGI1* insulin-like growth factor-1, *Bnip3* Bcl-2 binding and pro-apoptotic protein 3, *BCAT2* branched-chain amino acid aminotransferase 2, *GAPDH* glyceraldehyde-3-phosphate dehydrogenase

### Measurement of the concentrations of higenamine and salsolinol in the decoctions of PA commercial products

Samples of commercial PA products (PA1, PA2, and PA3 of Japanese Pharmacopoeia XVIII Edition grade and the dried tuberous root of *A. carmichaelii* or *A. japonicum*, prepared by the processing described above, were purchased from Uchida Wakanyaku, Sanwa Shoyaku (Tokyo, Japan), Tsumura (Tokyo, Japan), Matsuura Yakugyo (Nagoya, Japan), and Tochimoto Tenkaido (Osaka, Japan). Table 2 presents the sample lists with the names of the distributers and lot numbers. Unprocessed aconite root, the dried tuberous root of *A. carmichaelii*, was purchased from Tochimoto Tenkaido (lot number, Lot. 32009004). Some samples were supplied as small pieces by cutting the whole crude drug into 2–4 mm blocks. The samples (about 30 g) were powdered using a mill, and passed through a sieve (300 µm). The powdered samples (25 mg) were mixed with 0.50 ml of ion-exchanged water and heated at 100 °C for 30 min. Following the centrifugation (1.2 × 10^4^×*g*, 5 min), the supernatant was kept at − 20 °C until analysis. A 15 µl aliquot of the supernatant was mixed with 135 µl of 1% formic acid and 30 µl of methyllycaconitine (Santa Cruz Biotechnology, Dallas, TX, USA, 34 µg/ml) in 1% formic acid for use as an internal standard. Following centrifugation (1.2 × 10^4^×*g*, 10 min), the supernatant was transferred into a glass vial for LC–MS/MS system (Waters Quattro Premier XE, Milford, MA, USA) with an electrospray ionization source in the positive ion mode and multiple reaction monitoring. HPLC separation was performed under the following conditions: column, Waters Acquity UPLC HSS C18 1.8 µm, 2.1 × 100 mm; mobile phase, linear gradient elution system, 0.05% AcOH in H_2_O (solvent A): 0.05% AcOH in acetonitrile (solvent B) (A/B) = 99/1–80/20 for 0–1 min; 80/20–50/50 for 1–2 min; 50/50–15/85 for 2–3.5 min; 15/85–99/1 for 3.5–3.6 min; 99/1 for 3.6–6.5 min at a flow rate of 0.2 ml/min. The injection volume of the sample was 10 µl. Both quadrupoles were maintained at the unit resolution and the transitions (precursor to daughter) monitored were 179.9 → 162.9 *m/z* for salsolinol (retention time, 2.4 min), 272.0 → 106.8 *m/z* for higenamine (2.9 min), and 683.4 → 108.4 *m/z* for methyllycaconitine (3.4 min). Linear regressions of the concentration ranges of salsolinol and higenamine were calibrated by the peak area ratio of these compounds to methyllycaconitine using the least-squares method (*r*^2^ > 0.99).

**Table 2:** Concentrations of salsolinol and higenamine in the decoctions of commercially available processed aconite root (PA) or unprocessed aconite root (uzu)

	Vendor	Lot #	Salsolinol	Higenamine
PA1	Uchida	262114	20 ± 5	0.18 ± 0.11
PA1	Uchida	C4S0243	13 ± 2	0.046 ± 0.017
PA1	Uchida	D8K0243	23 ± 11	0.077 ± 0.022
PA1	Uchida	D8K0S19	27 ± 11	0.059 ± 0.005
PA1	Uchida	E850243	25 ± 15	0.24 ± 0.08
PA1	Uchida	F2F0243	15 ± 1	0.10 ± 0.02
PA1	Sanwa	–	16 ± 5	0.15 ± 0.00
PA1	Tsumura	K04331	18 ± 2	0.40 ± 0.06
PA1	Matsuura	H6L1	19 ± 8	0.26 ± 0.02
PA2	Uchida	E9H0519	3.5 ± 0.7	0.014 ± 0.003
PA2	Tochimoto	180804	5.2 ± 2.0	0.023 ± 0.006
PA2	Tochimoto	31415001	4.8 ± 2.4	0.022 ± 0.003
PA3	Uchida	E7S0514	0.24 ± 0.19	n.d.
Uzu	Tochimoto	180804	4.9 ± 1.0	0.011 ± 0.005
	Mean ± SD		14 ± 9	0.12 ± 0.12
	CV (%)		63%	97%

PA1, PA2, and PA3 were defined in processed aconite root (PA) section in Japanese Pharmacopoeia 18th Edition [22]. Each dried sample was powdered by mill, and each powder (25 mg) was decocted with 0.50 ml water for 30 min, then, centrifuged (15,000 rpm for 5 min), and the supernatant was analyzed using LC–MS/MS. "n.d." for higenamine means less than 0.0017 µg/ml. Each data (µg/ml) is the mean ± SD for 3 batches of the decoction in each lot. CV, coefficient of variation

### Statistical analysis

One-way analysis of variance (ANOVA) followed by Bonferroni's multiple test was used to compared multiple data. Data are expressed as mean ± standard error (SE), and *P* < 0.05 considered significant. All analyses were conducted using Mac Statistic Analysis Ver 3.0 (Esumi, Tokyo, Japan).

## Results

**Fig. 1:**
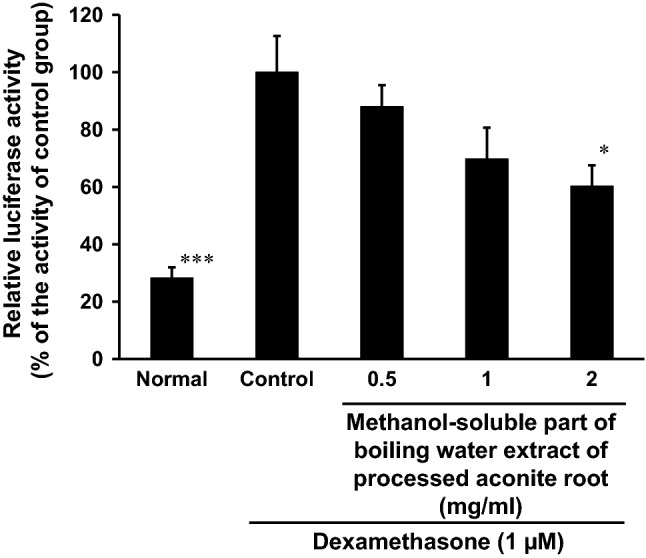
Effect of processed aconite root (PA) extract on dexamethasone-induced MuRF1 promoter expression in differentiated C2C12 cells. Differentiated C2C12 cells were transfected with pGL3-MuRF1 and pCMVβ-gal plasmids, and treated with or without dexamethasone (1 µM) and the methanol-soluble part of boiling water extract of PA for 24 h. Control group was treated with dexamethasone without the extract. Cell lysates from cells were used in luciferase assay. Data are mean ± SD (*n* = 5). *P < 0.05 and ***P < 0.001 compared with control group by Bonferroni's multiple tests.

Dexamethasone-induced MuRF1 promoter expression in differentiated C2C12 cells at 1 µM was significantly suppressed by methanol-soluble part of the boiling water extract of PA in a concentration-dependent manner with its IC_50_ value of 1.5 mg/ml (Fig. 1). By MTT assay, no cytotoxicity was observed by the concentration of 2.0 mg/ml (data not shown).

**Fig. 2:**
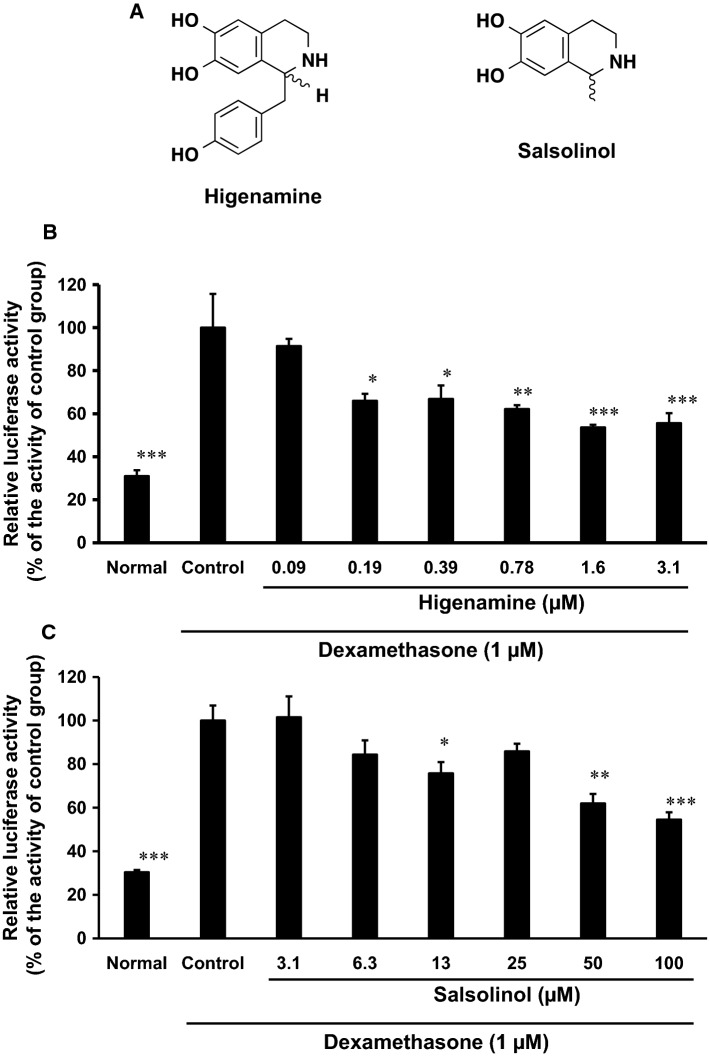
Effect of higenamine and salsolinol on dexamethasone-induced MuRF1 promoter expression in differentiated C2C12 cells. **A** Chemical structure of higenamine and salsolinol. **B**, **C** Differentiated C2C12 cells were transfected with pGL3-MuRF1 and pCMVβ-gal plasmid and treated with or without 1 µM dexamethasone and higenamine (**B**) or salsolinol (**C**) for 24 h. Control group was treated with dexamethasone without higenamine or salsolinol. Lysates from cells were used in luciferase assay. Data are mean ± SD (*n* = 3). *P < 0.05, **P < 0.01, and ***P < 0.001 compared with control group by Bonferroni's multiple tests

Next, we tried to isolate the active ingredients by activity-guided fractionation. We obtained acidic layer, alkaline layer, BuOH layer, and water layer from PA as described in “Materials and methods”. Then, we evaluated the suppressive activities of them on dexamethasone-induced MuRF1 promoter expression and found that the activity was transferred into BuOH layer (data not shown). The BuOH layer was subjected to silica gel column chromatography, and the activity was transferred into Fr. 3 (data not shown). By preparative HPLC using ODS column, we isolated and identified salsolinol from Fr. 3 (Fig. 2A). Among the related compounds to salsolinol in other the constituents of PA, we obtained commercial reagent of higenamine [20]. Using commercial reagents, higenamine and salsolinol exhibited concentration-dependent suppressions on dexamethasone-induced MuRF1 promoter expression in differentiated C2C12 cells, and the IC_50_ values of higenamine and salsolinol were 0.49 and 50 µM, respectively (Fig. 2B, C).

We measured the contents of higenamine and salsolinol in the decoctions of commercially available thirteen kinds of PA products and one kind of unprocessed aconite root (Japanese name, *uzu*) product, and the data are shown in Table 2. The PA decoction used in the experiments mentioned above contained 0.10 and 15 µg/ml of higenamine and salsolinol, respectively. Their contents in fourteen products were varied and the coefficient of variation (CV) values were 97% and 63%, respectively.

**Fig. 3:**
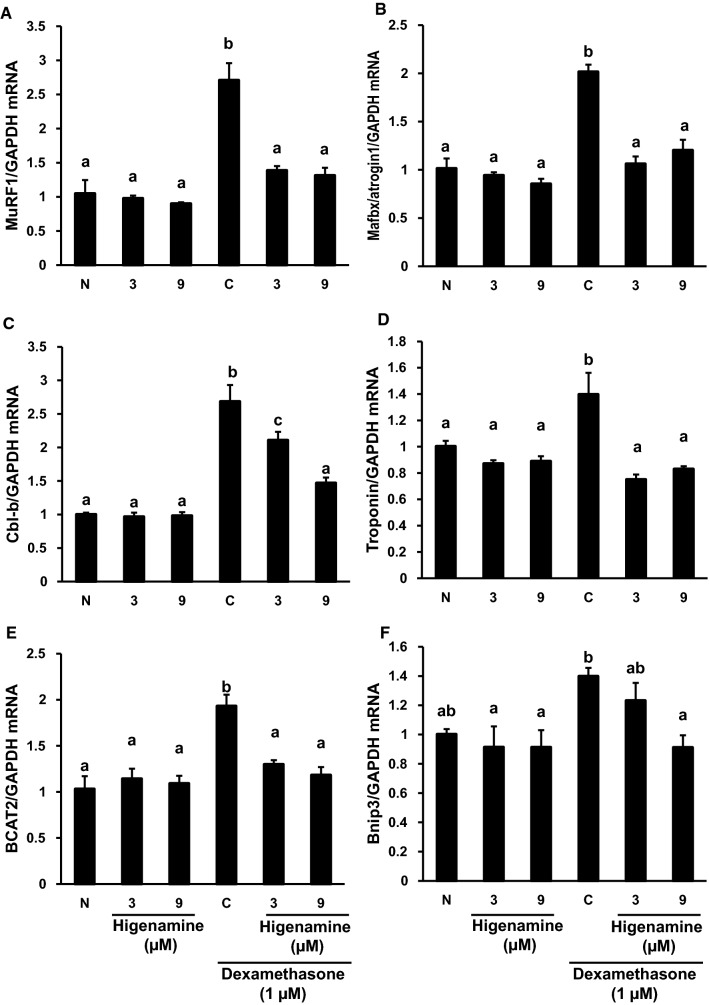
Effect of higenamine on mRNA expressions of muscle ring finger protein-1 (MuRF1) (**A**), muscle atrophy F-box protein (MAFbx)/atrogin 1 (**B**), casitas B-lineage lymphoma-b branched chain (Cbl-b) (**C**), troponin (**D**), branched-chain amino acid aminotransferase 2 (BCAT2) (**E**), Bcl-2 binding and pro-apoptotic protein 3 (Bnip3) (**F**) in differentiated C2C12 cells. Differentiated C2C12 cells were treated with the medium containing 1 µM dexamethasone and/or higenamine (3 or 9 µM) for 24 h, and total mRNA samples were collected and analyzed mRNA expressions of these genes using real-time PCR. Relative quantification of target gene was calculated using the − 2^ΔΔ*Ct*^ method, and data are expressed as fold changes of the target gene/glyceraldehyde-3-phosphate dehydrogenase (GAPDH) compared with those of the control. *N* the medium without dexamethasone and higenamine, *C* the medium containing dexamethasone (1 µM) without higenamine. Data are mean ± SD (*n* = 4). Different alphabetical letters a, b, and c over the columns in each figure panel indicate statistically significant differences at *P* < 0.05 evaluated by Bonferroni's multiple tests

We evaluated the suppressive effects of higenamine on MuRF1 mRNA expression. Although higenamine did not exhibit any inhibitions on MuRF1 mRNA expressions in normal condition of differentiated C2C12 cells, these compounds significantly suppressed dexamethasone-induced MuRF1 mRNA expressions (Fig. 3A). Higenamine also significantly suppressed dexamethasone-induced mRNA expressions of MAFbx/atrogin1 and casitas B-lineage lymphoma-b (Cbl-b), which are belonging to muscle-specific ubiquitin ligase similar to MuRF1, troponin which is integral to muscle contraction in skeletal and cardiac muscle, branched-chain amino acid aminotransferase 2 (BCAT2) that attenuates muscle protein degradation, and Bcl-2 binding and pro-apoptotic protein3 (Bnip3) that is the marker of autophagy. The suppression on Cbl-b and Bnip3 exhibited concentration-dependent manners (Fig. 3B–F). Dexamethasone significantly suppressed mRNA expressions on myosin heavy chain (MyHC) and insulin-like growth factor-1 (IGF1), but higenamine at 3.0 and 9.0 µM did not exhibited significant effect on theses suppressions (data not shown). Higenamine at 3.0 and 9.0 µM did not exhibit any effects on the mRNA expressions of these target genes in C2C12 cells without dexamethasone treatment.

## Discussion

The root of *Aconitum carmichaelii* is a well-known crude drug to relieve pain related to cold symptoms [14]. Its raw root contains toxic diterpene alkaloids, such as aconitine and mesaconitine, LD_50_ values of which are 0.5–1.8 g/kg for oral administration in mice [21]; therefore, various processing methods to reduce the toxicity of the root of *Aconitum carmichaelii* have been developed, and the eighteenth edition of the Japanese Pharmacopoeia (JPXVIII) registers the dried material of the autoclaved root of *A. carmichaelii* as the item name processed aconite root (PA) [22]. Highly toxic diterpene alkaloids are degraded into less toxic diterpene alkaloids (*e.g.*, benzoylmesaconine) by heating or autoclaving [21], and the toxicity of benzoylmesaconine is about one-eight hundredth of mesaconitine [23]. Then, PA is considered to be a safe and effective herbal agent to relieve pain. Although the analgesic activity of benzoylmesaconine in tail pinch test using mice was much less than that of mesaconitine about one-thousandth [24], neoline remained in PA after the heat processing of *A. carmichaelii* raw root to exhibit the effectiveness for neuropathic pain [15, 16].

In the present study, we evaluated the effectiveness of PA related to another traditional knowledge of the effectiveness to supply the *kidney* energy [14]. In traditional Kampo medicine and traditional Chinese medicine, the deficiency of *kidney* energy is related to aging, and several diseases associated with old age, such as frequent urination, lumbar pain, lethargy, blurred vision, sarcopenia, and frailty [3, 25]. Therefore, we explored the effectiveness of PA to prevent muscular atrophy and evaluated its suppressive effect on dexamethasone-induced MuRF1 expression in C2C12 cells in vitro. Then, we found that it was significantly suppressed by methanol-soluble part of boiled water extract of PA with its IC_50_ value of 1.5 mg/ml. This titer was weaker than the effect of PA boiled water extract on oxaliplatin-induced reduction of neurite elongation in dorsal root ganglion neurons (the effective concentration, 0.1 mg/ml) [15].

In the process of activity-guided fractionation of PA extract, we found that the active ingredients of the suppressive effect on dexamethasone-induced MuRF1 expression did not transfer into the fraction containing aconitine-type diterpene alkaloids, the major pharmacologically active ingredients of PA [15, 16, 24]. Then, we finally found higenamine and salsolinol as the active constituents in PA in the fraction containing relatively hydrophilic and neutral compounds. Since higenamine and salsolinol are alkaloidal compounds but they have both phenolic hydroxyl groups and amine motif in their chemical structures, it can be reasonable to find these compounds in this fraction. Higenamine was isolated from the raw root of *A. japonicum* as the cardiac tonic principle [20], and salsolinol was isolated from the raw root of *A. carmichaelii* [17].

We measured the contents of higenamine and salsolinol in the decoctions of available PA or unprocessed aconite root products (*uzu*). When 25 mg of PA sample used in the first experiments was decocted in 0.5 ml of water, the decoction contained 0.10 and 15 µg/ml of higenamine and salsolinol, respectively. By these data, the contents of higenamine and salsolinol in PA sample were 0.00020 (w/w) % and 0.030 (w/w) %, respectively. The IC_50_ of the methanol-soluble part of boiling water extract of PA (1.5 mg/ml) can be converted to 12 mg/ml of PA by the ratio yielded, and this concentration was further converted to 23 ng/ml (= 0.086 µM) of higenamine and 3.5 µg/ml (19 µM) of salsolinol. Using the IC_50_ values of higenamine and salsolinol at 0.49 and 50 µM, respectively, it is considered that higenamine and salsolinol contributed the effectiveness of PA about 18% and 38%, respectively. The contents of salsolinol and higenamine were variated among the commercially available PA products. The contents of salsolinol and higenamine in PA3 product were the lowest among PA products analyzed, and those in PA2 were tended to be lower than those in PA1. The processing method of PA3 was the treatment with calcium hydroxide after rinsing in salt solution, and that of PA2 was heating or autoclaving after rinsing in salt or rock salt solution [22]. Since salsolinol and higenamine are hydrophilic compounds, they may easily be extracted and lost in the process of rinsing in salt solution. The contents of salsolinol and higenamine in *uzu* were similar to those of PA2 products of the same pharmaceutical company, suggesting that the contents of salsolinol and higenamine may not change by heat processing and may play as the active ingredients of PA after heat processing, like neoline [16].

Since the titer of higenamine in the present activity was about 100-fold higher than that of salsolinol, the further pharmacological evaluations were focused on higenamine. Dexamethasone stimulates glucocorticoid receptor in skeletal muscle to activate Krüppel-like factor 15, that promotes the expressions of MuRF1, MAFbx/atrogin-1, and Cbl-b to induce ubiquitin–proteasome-dependent protein degradation and muscle atrophy, the expression of Bnip3 to induce autophagy, and the expression of BCAT2 to induce the feedback system and to suppress the function of glucocorticoid receptor [26]. Since higenamine did not exhibit any effects on the mRNA expressions of MuRF1, MAFbx/atrogin-1, Cbl-b, Bnip3, BCAT2, and troponin in normal condition of differentiated C2C12 cells, higenamine did not affect the homeostasis of C2C12 cells. However, higenamine significantly suppressed their mRNA expressions stimulated by dexamethasone; therefore, higenamine has the protective effects against the violation of dexamethasone in differentiated C2C12 cells. Although the molecular target of higenamine to protect dexamethasone-induced muscle atrophy is unknown, higenamine did not affect the signal transduction from IGF1 receptor stimulation to myosin heavy chain expressions but that from glucocorticoid receptor stimulation into skeletal muscle atrophy in the catabolic processes, since higenamine did not counteract the suppression of MyHC and IGF1 mRNA suppressions induced by dexamethasone in differentiated C2C12 cells.

Higenamine is well-known β_2_-adrenoceptor agonist and is registered in the World Anti-Doping Agency (WADA) Prohibited Substances and Methods list [27]. Higenamine has vasodilating and anti-inflammatory effects on aorta [28, 29], anti-aggregating activity on platelets [30], and anti-apoptotic effects on hypoxia-induced brain injury [31, 32]. Higenamine protects the cardiac injury induced by ischemia/reperfusion, collagen-induced arthritis, and the apoptosis gastric smooth muscle cells in diabetes via the activation of β_2_-adrenoceptor and phosphoinositide 3-kinase (PI3K)/protein kinase B (AKT) signaling pathways [33–35]. On the other hand, trimetazidine, an anti-anginal agent, significantly counteracted dexamethasone-induced skeletal muscle atrophy and the phosphorylation of PI3K and AKT in C2C12 cells, suggesting that dexamethasone would induce skeletal muscle atrophy by the suppression of PI3K/AKT signaling pathways [36]. Considering the above results together, higenamine might suppressed dexamethasone-induced muscle atrophy by activating β_2_-adrenoceptor and PI3K/AKT signaling pathways.

When higenamine (50 mg/kg) was orally administered into rabbits, the maximum blood concentration was 2.9 µg/ml appeared at 10 min after the administration [37]. When 3 g of PA1 sample (Tsumura, K04331) was decocted in 60 ml of water, about 24 µg of higenamine can be collected, and the dosage in human is calculated as 0.48 µg/kg. By this dosage and the result of pharmacokinetic study in rabbits [37], the maximum blood concentration of higenamine by taking PA1 (3 g) in human can be calculated as 28 pg/ml. Since this estimated blood concentration of higenamine was much lower than its IC_50_ value (0.10 µg/ml) in the present study, the contribution of higenamine as the active ingredients in PA to the prevention from muscular atrophy could be small in clinic, and further studies to find other active ingredients in PA were demanded.

The pharmacology of PA related to the effectiveness for the deficiency of *kidney* energy in traditional Japanese Kampo medicine and traditional Chinese medicine might be the protective effects on muscular atrophy, and we found higenamine and salsolinol as the active ingredients. Although the clinical contribution of these compounds to the prevention from muscular atrophy is not high, these compounds can be considered as the active ingredients of PA and be used as the marker compounds for the quality control of PA.

## Data Availability

The data used to support the findings of this study are available from the corresponding author upon request.
