# Correction to: Suppressive effects of processed aconite root on dexamethasone-induced muscle ring finger protein-1 expression and its active ingredients

**DOI:** 10.1007/s11418-023-01691-0

**Published:** 2023-03-16

**Authors:** Taishi Kondo, Tomoaki Ishida, Ke Ye, Marin Muraguchi, Yohei Tanimura, Masato Yoshida, Kan’ichiro Ishiuchi, Tomoki Abe, Takeshi Nikawa, Keisuke Hagihara, Hidetoshi Hayashi, Toshiaki Makino

**Affiliations:** 1 Department of Pharmacognosy, Graduate School of Pharmaceutical Sciences, Nagoya City University, 3-1 Tanabe-Dori, Mizuho-ku, Nagoya, Aichi 467-8603 Japan; 2 Healthy Food Science Research Group, Cellular and Molecular Biotechnology Research Institute, National Institute of Advanced Industrial Science and Technology (AIST), 1-1-1 Higashi, Tsukuba, Ibaraki 305-8566 Japan; 3 Department of Nutritional Physiology, Institute of Medical Nutrition, Tokushima University Graduate School, 3-18 Kuramoto-cho, Tokushima, 770-8503 Japan; 4 Department of Advanced Hybrid Medicine, Graduate School of Medicine, Osaka University, 2-2 Yamadaoka, Suita, 565-0871 Japan; 5 Department of Cell Signaling, Graduate School of Pharmaceutical Sciences, Nagoya City University, 3-1 Tanabe-Dori, Mizuho-ku, Nagoya, Aichi 467-8603 Japan

**Correction to: Journal of Natural Medicines (2022) 76:594–604** 10.1007/s11418-022-01606-5

The article Suppressive effects of processed aconite root on dexamethasone induced muscle ring finger protein 1 expression and its active ingredients, written by Taishi Kondo, Tomoaki Ishida, Ke Ye, Marin Muraguchi, Yohei Tanimura, Masato Yoshida, Kan’ichiro Ishiuchi, Tomoki Abe, Takeshi Nikawa, Keisuke Hagihara, Hidetoshi Hayashi, Toshiaki Makino, was originally published Online First without Open Access. After publication in volume 76, issue 3, page 594–604 the author decided to opt for Open Choice and to make the article an Open Access publication. Therefore, the copyright of the article has been changed to © The Author(s) 2023 and the article is forthwith distributed under the terms of the Creative Commons Attribution 4.0 International License, which permits use, sharing, adaptation, distribution and reproduction in any medium or format, as long as you give appropriate credit to the original author(s) and the source, provide a link to the Creative Commons licence, and indicate if changes were made. The images or other third party material in this article are included in the article’s Creative Commons licence, unless indicated otherwise in a credit line to the material. If material is not included in the article’s Creative Commons licence and your intended use is not permitted by statutory regulation or exceeds the permitted use, you will need to obtain permission directly from the copyright holder. To view a copy of this licence, visit http://creativecommons.org/licenses/by/4.0/.

The original article has been updated.

